# Establishing and sustaining research partnerships in Africa: a case study of the UK-Africa Academic Partnership on Chronic Disease

**DOI:** 10.1186/1744-8603-8-29

**Published:** 2012-08-16

**Authors:** Ama de-Graft Aikins, Daniel K Arhinful, Emma Pitchforth, Gbenga Ogedegbe, Pascale Allotey, Charles Agyemang

**Affiliations:** 1 Regional Institute for Population Studies, University of Ghana, Legon, Accra, Ghana; 2 LSE Health, London School of Economics and Political Science, London, UK; 3 Noguchi Memorial Institute for Medical Research, University of Ghana, Legon, Ghana; 4 RAND Europe, Cambridge, UK; 5 School of Medicine, New York University, New York City, NY, USA; 6 School of Medical and Health Sciences, Monash University Sunway Campus, Bandar Sunway, Malaysia; 7 Amsterdam Medical Centre, University of Amsterdam, Amsterdam, Netherlands

## Abstract

This paper examines the challenges and opportunities in establishing and sustaining north–south research partnerships in Africa through a case study of the UK-Africa Academic Partnership on Chronic Disease. Established in 2006 with seed funding from the British Academy, the partnership aimed to bring together multidisciplinary chronic disease researchers based in the UK and Africa to collaborate on research, inform policymaking, train and support postgraduates and create a platform for research dissemination. We review the partnership’s achievements and challenges, applying established criteria for developing successful partnerships. During the funded period we achieved major success in creating a platform for research dissemination through international meetings and publications. Other goals, such as engaging in collaborative research and training postgraduates, were not as successfully realised. Enabling factors included trust and respect between core working group members, a shared commitment to achieving partnership goals, and the collective ability to develop creative strategies to overcome funding challenges. Barriers included limited funding, administrative support, and framework for monitoring and evaluating some goals. Chronic disease research partnerships in low-income regions operate within health research, practice, funding and policy environments that prioritise infectious diseases and other pressing public health and developmental challenges. Their long-term sustainability will therefore depend on integrated funding systems that provide a crucial capacity building bridge. Beyond the specific challenges of chronic disease research, we identify social capital, measurable goals, administrative support, creativity and innovation and funding as five key ingredients that are essential for sustaining research partnerships.

## Introduction

There has been a growing number of research partnerships between high and low-income regions over the last two decades [1]. The structures of partnerships are dependent on the funding organization, the empirical and geographical focus of the research, the disciplines involved and the research capacity of collaborating institutions or groups. This has led to various permutations involving ‘north-south’ or ‘south-south’ collaborations with ownership centred either within the northern institution, southern institution or shared between both [1-4]. In an era of globalization with increased connectivity between countries and complex developmental challenges, there is a consensus that research partnerships must play an important role in knowledge production and the development of global solutions [1-4]. However, an underlying theme in recent reflections on how research partnerships work is the difficulty of sustaining and scaling up short-term achievements because of complex micro-political (e.g. power struggles between members) and macro political (e.g. the demands of the funding organization) processes [1-3,5]. It has become important to record and reflect on the dynamics of partnerships as a learning process for existing and future partnerships, particularly those situated within or led from low-income and low research capacity countries [1,3,6].

In this paper we present a case study of an African-centred north–south research partnership led by a northern institution. The UK-Africa Academic Partnership on Chronic Disease (hereafter the Partnership), was established in 2006 with seed funding from the British Academy. It aimed to address chronic non-communicable diseases (hereafter NCDs or chronic diseases) research, practice and policy for the sub-Saharan African region and for sub-Saharan Africans in Europe.

Africa has a complex disease burden. Infectious diseases such as malaria, tuberculosis and HIV/AIDS, neglected tropical diseases like onchocerciasis and schistosomiasis, and nutritional disorders are major causes of disability and death [7]. At the same time prevalence rates of chronic non-communicable diseases (NCDs) such as cardiovascular diseases, cancers and diabetes, are increasing. While 70% of deaths in Africa can be attributed to infectious diseases, NCD deaths in men and women as a whole are higher in sub-Saharan African than in virtually all other regions of the world [8]. African health systems are weak and cannot cope with the cumulative burden of infectious and chronic diseases. There are inadequate numbers of NCD specialists, health facilities are ill-equipped, medicines are either inaccessible or expensive, and few countries have developed policies to address chronic disease care [9-11]. Over the last ten years, health governance and financing on the continent has been influenced by the growing number of Global Health Initiatives (GHIs) and the quest to attain the Millennium Development Goals. This has had a major impact on how governments, health policymakers and researchers set and tackle local health priorities. At least 80% of health financing has focused on infectious disease and capacity for NCD care is weak [12]. Similarly, health research in many countries focuses predominantly on infectious diseases, and particularly on HIV/AIDS [7,10]. Yet, NCDs, as UN Secretary General Ban Ki-Moon has argued, constitute ‘a public health emergency in slow motion’ [13], and continued neglect of their public health impact will significantly undermine attainment of the MDGs [13,14]. Since the publication of the 2005 WHO report, *Preventing Chronic Diseases. A vital investment*[15], there has been increasing emphasis on the urgent need for creative and cost-effective solutions in the African region and a strong consensus that multi-stakeholder partnerships can produce these kinds of solutions [14,16]. The idea for the chronic disease partnership was developed within this context.

African-centred chronic disease partnerships exist, although they are few in number compared to infectious disease partnerships in the region and chronic disease initiatives in Europe and North America. The majority are funded and led by Euro-American institutions. Some chronic disease partnerships in Africa have focused on single chronic conditions in single countries, for example diabetes in Ghana [17] or Cameroon [18]. Others have focused on single conditions across a number of African countries or across the global context with representative African countries. Key examples include the mental health and poverty project (MHaPP), which focuses on Ghana, Zambia, Uganda and South Africa [19] and the International of Asthma and Allergy in Childhood (ISAAC) Phase II, which gathered data on asthma in children across 30 sites in 22 countries including rural Ghana [20].

There is an emerging trend of African-centred chronic disease partnerships publishing their experiences. Recent papers have focused on a UK-Cameroon partnership to establish a national diabetes care model [18], a UK-Botswana partnership on public health, including a focus on epilepsy [21] and on a UK-Ghana partnership on stroke care [22].

The partnership, described in full below, was different from existing chronic disease partnership at three levels. First, we focused on a range of important chronic diseases across a number of African countries, as well as for African communities living in Europe. Secondly, as part of our annual reporting commitments to our funder, The British Academy, the lead partners had to monitor and evaluate progress against our goals—therefore reflexivity was built into project management from the beginning of the project. Finally, we had limited funding which prevented collaborative research. In the following sections we present criteria against which the achievements of any research partnership may be evaluated and then apply these in a detailed case study of the Partnership. We identify the elements required for sustaining successful chronic disease research partnerships within a regional context where infectious disease research, practice and policy are prioritised. We then reflect on the broader lessons for research partnerships.

## Conceptual framework

**Figure 1:**
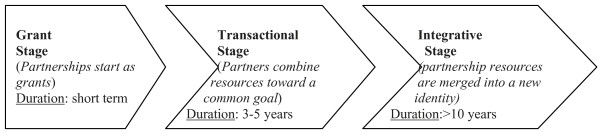
** The Collaborative Continuum.** Source: Adapted from McRobbie and Kolbe, 2009 [24] and Whitworth et al., 2008 [3].

A number of frameworks exist for evaluating the process and outcomes of partnerships. Some focus on multi-stakeholder collaborations such as partnerships between donors, governments and research institutions [3], others focus on academic or research partnerships [2,5,23]. Our evaluation of the successes and challenges of the Partnership is informed by eleven key principles of successful research partnerships outlined by Maselli et al. (2005) [5] (see Table 1). The Maselli et al. conceptual framework focuses on research partnerships. Its principles are captured in other discussions of establishing and sustaining research partnerships, particularly within the African context [6]. We also draw on broader discussions within public health on the ‘collaboration continuum’ where partnerships thrive through a “balanced division of labor and resources” that might begin “as a grant at one end; progress to a “transactional stage” in which partners combine resources toward a common goal; and culminate in an “integrative stage” characterized by merging resources to generate a new identity” (McRobbie and Kolbe, 2009, p. 3) [24] (Figure 1). Similar three-stage models are proposed elsewhere, with the consensual assumption that partnerships start small with seed funding and graduate through internal creativity and external financial support to large-scale long-term projects or institutions [3,6]. The concept of a ‘collaborative continuum’ is essential to our analysis because it places emphasis on a project in progress and of the importance of understanding the fine balance of ‘labour and resources’ that makes the partnership work at each conceptual and/or operational stage.

**Table 1:** Criteria for developing successful communities of research excellence

	
1.	Decide on objectives together
2.	Build up mutual trust
3.	Share information, develop networks
4.	Share responsibility
5.	Create transparency
6.	Monitor and evaluate collaboration
7.	Disseminate the results
8.	Apply the results
9.	Share profits equitably
10.	Increase research capacity
11.	Build on achievements

Source: (Masselli, Lys and Schmid (2005) [5]

## The UK-Africa academic partnership on chronic disease: background

The UK-Africa Academic Partnership on Chronic Disease was established in 2006 with funding of £29,166 from the British Academy’s inaugural UK-Africa Academic Partnerships (see http://www.britac.ac.uk/funding/awards/intl/africapartnerships.html). The project was conceptualized and drafted by the first author (AdGA), developed by the first and second (DKA) authors, and fine-tuned through consultation with a group of 15 UK and Ghanaian academics. It was developed specifically for the British Academy’s call for grant proposals for UK-Africa academic networks based on ‘themes of common interest’. The project was co-owned by the authors’ institutions: University of Cambridge (AdGA) and University of Ghana (DKA). However, the British Academy stipulated that the UK-Africa academic partnerships grants would be held and managed by the UK principal partner’s institution. Operationally, the grant—of £30,000 or less to be spent over three years—could not fund original collaborative research.

The initial proposal group came from different disciplines but all members conducted research on chronic disease in Ghana, West Africa and South Africa or in the UK among African or Asian populations (see Table 2 ). 16 members had worked or networked together in various capacities prior to the start of the project. Long-term friendships and working relationships existed between a subset of this group. AdGA and DKA, for example, had a ten year working relationship, which included research on diabetes and hypertension in Ghana in the late 1990s [25]. Similar long-term working relationships existed between London-based and Accra-based researchers. Social familiarity, openness and trust were key components of the development process.

**Table 2:** Evolution of the partnership, 2006—2010

**End of Year 1 (2007)**	**End of Year 2 (2008)**	End of Year 3* (2010)
Burkina Faso (1)	Cameroon (3)	Cameroon (3)
*(Med - 1)*	*(Anth-1; Ling -1; Psy-1)*	*(Anth-1; Ling -1; Psy-1)*
Ghana (10)	Ghana (12)	Ghana (13)
*(Anth-1; B.Sci -1;Geog-1; Psy-2; Med-4; Nutr-1)*	*(Anth-1; B.Sci -1;Geog-2; Psy-2; PubH-1; Med-4; Nutr-1)*	*(Anth-1; B.Sci -1;Geog-2; Pol-1; Psy-2; PubH-1; Med-4; Nutr-1)*
Nigeria (2)	Kenya (1)	Kenya (1)
(*Med -2*)	*(PubH-1)*	*(PubH-1)*
UK (10)	The Netherlands (1)	The Netherlands (1)
*(Anth-1; Med-2; Nutr-1; Psy-5; Soc-1)*	*(PubH-1)*	*(PubH-1)*
Total number: 23	Nigeria (3)	Malaysia (1)
	*(Med-3)*	*(Anth-1)*
	South Africa (1)	Nigeria (4)
	*(H.Sci-1)*	*(Med-3; Pol-1)*
	UK (14)	South Africa (1)
	*(Anth-1; Hsci-1;Med-4; Nutr-3; Psy-5)*	*(H.Sci-1)*
	Total Number: 35	UK (15)
		*(H.Sci-1;Med-5; Nutr-3; PubH-1; Psy-5)*
		US (3)
		*(Med-1; HSci-2)*
		Total Number: 42

**Key:** Anth – Anthropology; B.Sci – Biological Sciences; Geog – Geography; H.Sci – Health Sciences; Med – Medicine (including Psychiatry); Nutr- Nutrition; Pol – Policy; Psy – Psychology; PubH – Public Health; Soc – Sociology;

**Note:** Profile excludes postgraduate student members from Ghana (5), UK (5), Denmark (2) and the US (1) who joined in Years 1 and 2.

Our theme of common interest was ‘Africa’s neglected chronic disease burden’. We made a theoretical and empirical case for the necessity of an interdisciplinary network on chronic disease based on the three well-documented challenges in chronic disease research that are linked to the broader political economy of health research in Africa:

1. the recognition that Africa had a significant NCD burden [15],

2. the fact that the chronic disease burden was a complex public health problem given its co-existence with a significant burden of infectious communicable diseases [9], and

3. the consensus on a need for interdisciplinary research and for particular contributions from the social sciences and humanities [9,15,26].

The partnership had four goals:

1. To integrate social and biomedical science research on chronic diseases of public health significance in Africa and for African communities in the UK through collaborative research between partners;

2. To influence chronic disease policies in Africa by disseminating evidence-based research and intervention to policy makers;

3. To offer postgraduate teaching, training and support in Africa and in the UK on Africa-centred chronic disease through teaching exchanges, research internships and co-publishing;

4. To disseminate partnership research and related activities to academic, health practitioner/policy and lay audiences through a partnership website, annual meetings and joint publications.

These goals were refined over the partnership’s four-year history in response to logistical and professional challenges (see following sections).

### Structure

We began with 17 members, including the UK and Africa principal partners. We aimed for an interdisciplinary structure with the social sciences dominating to redress the medical dominance in chronic disease research in the region (see Table 1 and Table 2). During the period of funding (2006–2010) the network expanded to include members from West, East and South Africa, as well as from Europe and the United States (see Table 2). We maintained the interdisciplinary focus and secured the membership of humanities scholars and of policymakers. We also lost some of our initial members due to their changing professional circumstances. Of the original 17 members named on the initial grant application, only 9 remained actively involved in partnership activities by March 2010, and only 5 of the original group were members of the core working group.

### Measuring engagement

At the start of the project the lead partners (AdGA and DKA) set up three levels of engagement based on their knowledge of the challenges in managing research partnerships in Ghana. By offering flexibility in participation and tracking members’ participation at clearly defined levels of engagement we aimed to facilitate the process of monitoring and evaluating project activities and goals, as well as to fulfill our annual reporting commitments to the British Academy. These levels of engagement were presented and discussed at our first workshop in Accra in 2007 where 60% of members (n = 23) were present (see Table 3).

· Level 1, the lowest, involved committing to at least one goal over the funded life of the project. This could include: (1) attending and/or presenting at one annual meeting (Goal 2 and 4); or (2) committing to one non-international travel teaching exchange in the life of the project (e.g. a UCL based partner teaching at LSE; a KNUST researcher teaching at the University of Ghana, Legon) (Goal 3); or (3) contributing to one project publication (Goal 1).

· Level 2 involved committing to at least two goals: (1) presenting at one or more meetings (Goals 2 and 4); and (2) committing to at least one teaching exchange (international or non-international) (Goal 3), or contributing to one project publication (Goal 1).

· Level 3 involved committing to at least three goals. It was envisaged that partners committed to Level 3 would form the core working group around whose empirical work the project would be built over the three years of BA funding. It was also envisaged that as part of Goal 1, this working group would collaborate on grant proposals to secure funding to scale up partnership activities post BA funding.

**Table 3:** Level of Engagement of Partnership members, 2007–2010

**Activity**	**Total membership**	**Percentage participation**
1^st^ Annual workshop (Accra, 2007)	23	14 (60%)
2^nd^ Annual workshop (LSE, 2008)	30	14 (47%)
BA/RS/GAAS International Conference (Accra, 2009)	35	15 (43%)
Monash Chronicity Conference (Kuala Lumpur, 2010)	42	9 (21%)
*Globalization and Health* Special Issue (2009/2010)	42	17 (40%)
*Ghana Medical Journal* Special Issue (2009-2010)	42 [24 Ghana experts]	10 [of 24 experts] (42%)
Grant applications (2008 – 2010)	42	10 (24%)

Based on the levels of engagement in key project activities recorded over the funded life of the project (see Table 4), about 23% of the partnership (10 out of the total membership of 44) could be categorised as active members, the ‘core working group’ that was committed to the vision of the partnership and contributed valuable time, skills and resources to the activities and development of the partnership (Table 3). The remaining constituted a mix of supportive members (average to low engagement) and passive members (low to no engagement).

**Table 4:** Core working group and individual contributions

	**Goal 1**	**Goal 2**	**Goal 3**	**Goal 4**
Juliet Addo	+++	+++	−	+++
Charles Agyemang	+++	+++	+++	+++
Pascale Allotey*	++	+++	−	+++
Kofi Anie*	++	+++	−	+++
Daniel Arhinful*	+++	+++	−	+++
Lem Atanga	+++	++	++	++
Catherine Campbell*	++	++	−	+++
Ama de-Graft Aikins*	+++	+++	+++	+++
Catherine Kyobutungi	++	++	++	+++
Olugbenga Ogedegbe	+++	+++	+++	+++
Emma Pitchforth	++	++	−	+++
Nigel Unwin	+++	+++	++	+++

Key: * members of the proposal group; +++ contributed to all aspects of the goal; ++ contributed to some aspects of the goal; - no contribution to the goalGoal 1: Publishing in the two special issues (at least 1); co-writing research proposals (at least 1 of 4)Goal 2: Dissemination of policy-relevant research through: (1) meeting presentations; (2) publications.Goal 3: Seminar lecture; research support; co-authoring publications with post-graduate studentsGoal 4: Attending annual meetings (at least 1); presenting/chairing at annual meetings (at least 1).

## A description of the partnership’s achievements and challenges

We describe the achievements and challenges across the four partnership goals.

### Goal 1: To integrate social and biomedical science research on chronic diseases of public health significance in Africa and for African communities in the UK through collaborative research between partners

Goal 1 was the most difficult to achieve during the funded period for two reasons. Our funding could not accommodate collaborative research and applications for funding to scale up our activities were unsuccessful.

Three grant applications were submitted to funders. The first two applications focused on developing chronic disease prevention interventions through participatory education. The first was submitted to Wellcome Trust in April 2008, with an African partner as Principal Investigator (PI) and three Africa-based and UK-based members as co-PIs. The second was submitted to ESRC in December 2008 with a UK-based PI, and seven Africa-based and Europe-based members as co-PIs and research consultants. Neither application was successful despite positive reviews from the Wellcome reviewers and a ‘good value for money’ endorsement from the ESRC reviewers. The third unsuccessful grant proposal was submitted to the Netherlands Organisation for Scientific Research (NWO) call for proposals for its Integrated Programme Scheme. The proposal was led by a Europe-based member, with four Africa-based and Europe-based members as co-PIs. Co-applicants for the three grants were all members of the core working group.

In light of these unsuccessful applications we adopted two creative strategies to develop active research projects. The first strategy was to draw on northern partners’ access to institutional funding for pilot projects. The first project was a collaboration in 2009 between Academic Medical Centre (AMC), University of Amsterdam (through last author CA) and African Population Health Research Centre (APHRC) (through core working group partner Dr Catherine Kyobutungi). The project focused on hypertension in a Nairobi slum. This resulted in the production of a University of Amsterdam Masters thesis. A manuscript on prevalence, awareness, treatment and control of hypertension in Nairobi, based on the thesis, is currently in preparation for journal publication. The second project was a collaboration in 2010 between the School of Medicine, New York University (NYU) (through fourth author GO) and the Regional Institute for Population Studies (RIPS), University of Ghana (through first author AdGA). The project examined the feasibility of establishing cardiovascular disease interventions in churches in Accra. Funding from the NYU Capstone Programme enabled four Global Health Masters students to conduct fieldwork with RIPS Masters students in Accra. The project led to an MPH project report and a co-authored publication in the *Bulletin of the WHO*[27].

The second strategy was to develop south-south collaboration through low-cost pilot studies that could be conducted in partner African countries and could feed into the postgraduate training goals as well as research proposals for funding. Two collaborations emerged from this. The first pilot project was conducted between partners based at the University of Ghana (UG) and focused on body image, perceptions and management in the UG community within the context of obesity and chronic diseases. Results of the project were presented at the 2010 UG Faculty of Social Studies Colloquium [28]. A manuscript is currently in preparation for journal publication. The second project was a collaboration between partners based at the University of Dschang, Cameroon and UG. The project examined media representations of chronic diseases in Ghana and Cameroon; postgraduate students at the University of Dschang assisted in gathering and analysing the data. Results of the project were presented at the 2010 UG Faculty of Social Studies Colloquium [29]; a manuscript has been submitted for peer review publication [30].

### Goal 2: To influence chronic disease policies in Africa by disseminating evidence-based research and intervention to policy makers

We adopted three strategies to achieve goal 2. First, we actively invited policymakers and members of donor agencies to all partnership workshops to participate as speakers or session chairs. Second, all workshop reports and proceedings were disseminated to policymakers based in Ghana, UK and Malaysia. Finally we actively sought the participation of policymakers in the production of our journal special issues, either as contributors or as reviewers. We secured the participation of policymakers and donors from the Ghana’s Ministry of Health, Ghana Health Service, Malaysia’s Ministry of Health, WHO-Ghana Office, WHO-Afro, WHO, UNAIDS-Nigeria Office, Royal Netherlands Embassy and UK’s DFID to our 2007 workshop and 2009 and 2010 international conferences. The 2007 workshop report was disseminated to Ghana’s Ministry of Health and Ghana Health Service. The *Ghana Medical Journal* special issue, which was informed by the 2007 Accra workshop, actively involved local policymakers as authors and reviewers. A local policymaker was invited to join the guest editorial team; he also contributed a review paper on trends and challenges in non-communicable disease policymaking in Ghana [31].

There was a major limitation to Goal 3. While we were able to disseminate our meeting outputs to local, regional and international policymakers, we were unable to monitor how the partnership outputs were used by policymakers. Limited funding prevented the monitoring and evaluation of processes external to the membership network.

### Goal 3: To offer postgraduate teaching, training and support in Africa and in the UK on Africa-centred chronic disease through teaching exchanges, research internships and co-publishing

Logistical and funding challenges led to minor restructuring of Goal 3. The focus on teaching exchanges had to be refined because UK and African universities operated different academic schedules and it was difficult to synchronise timetables of potential visiting lecturers and host institutions without incurring significant costs. We re-conceptualised teaching exchanges as opportunistic seminars, whereby UK or US partners visiting African countries on regular research activities were invited to give lectures or seminars at partner university departments. However, we were unable to facilitate these seminars, until after the funding period when two partners—one Europe-based, the other US-based—gave invited lectures at RIPS in 2011.

The workshops offered an alternative opportunity for postgraduate students to meet and network with partners. Therefore, we actively encouraged postgraduate participation in our workshops. Postgraduate student members conducting research on chronic diseases in Africa presented at partnership workshops in Ghana (NMIMR, 2007) and the UK (LSE, 2008).

The partnership facilitated two research internships: the aforementioned University of Amsterdam MSc research project based at APHRC, Nairobi and the NYU MPH group research project based at RIPS. Both internships were funded by the Northern institutions of the masters students with in-kind support from the African institutions.

The focus on co-publishing was the best realized strategy out of the three. We actively sought postgraduate contributions to our journal special issues and assisted some postgraduates in improving their papers for publication. Ten (10) postgraduate student members published papers in the two partnership flagship Special Issues in *Globalization and Health* (N = 4) and the *Ghana Medical Journal* (N = 7). Three postgraduates co-wrote their papers with partnership members. One postgraduate published in both issues. Of the 10 postgraduate students, 5 were based in Ghana and 5 in Europe. The NYU–RIPS collaboration, led to a co-publication between 2 core working group members, 4 NYU MPH students and 1 RIPS MPhil student [27].

### Goal 4: To disseminate partnership research and related activities to academic, health practitioner/policy and lay audiences through a partnership website, annual meetings and joint publications

Goal 4 yielded the greatest consistent success. We organised two major partnership workshops in Ghana (NMIMR, 2007) and London (LSE, 2008) and co-organised an international conference with Monash University in Malaysia (2010). In 2009 the partnership formed the basis for an international conference funded by the British Academy and the Royal Society in collaboration with the Ghana Academy of Arts and Sciences. These annual meetings attracted a large number of participants from diverse communities including researchers, practitioners, patient groups, policymakers and lay society. The workshops led to major publishing opportunities.

The 2008 partnership workshop led to an invitation to submit workshop proceedings as a special issue to the online open access journal *Globalization and Health*, by the journal’s co-Editor-in-Chief and fourth author, EP. The issue titled “Africa’s Disease Burden: local and global perspectives” was published in 2010 and guest edited by core working group members based in Africa, Asia and Europe [32]. Eight papers focused on major areas of Africa’s chronic disease burden including cardiovascular and risk factors among African migrant populations in Europe [33], co-morbid relationships between diabetes, tuberculosis and ART [8], diabetes experiences in Dar-es-Salaam [34], the social-cultural context of CVD in Africa [35], sickle cell disease [36], mental illness experiences and care in rural Ghana [37], care-giving in the context of HIV/AIDS in Kenya [38] and developing prevention strategies in Ghana and Cameroon [39]. Four (4) papers were based on original individual research by authors, with three stemming from postgraduate research; 4 papers were based on reviews conceptualized for the special issue. Twenty-nine (29) authors contributed to the issue: of these 6 were based in Africa, 16 based in Europe, 6 based in the US and 1 based in Asia. Two of the *Globalization and Health* publications [31,32] are highly accessed; each having been accessed more than 10,000 times.

A second special issue by the *Ghana Medical Journal* is in press. The issue titled “Ghana’s chronic non-communicable disease burden” is guest edited by core working group members based in Ghana, Europe and US. Ten papers submitted by members, including postgraduate students, focused on the epidemiology of asthma [40], hypertension [41] and stroke [42], the burden of mental illness [43], ageing and chronic diseases [44], experiences of type 1 diabetes [45] and terminal chronic conditions [46], household burden of chronic diseases [47], lay knowledge of the major chronic diseases [48] and local policy responses to the burden [31]. Five papers were based on original individual research; 5 papers were based on reviews conceptualized for the special issue. Nineteen (19) authors contributed to the issue: of these 11 were based in Ghana, 7 in the UK and 1 in the US.

Table 5 presents the levels of regional participation in our publishing projects. Out of a cumulative total of 46 authors, 42% were based in African institutions, 44% in European institutions and 11% in US institutions. Table 6 presents the disciplinary background of contributing authors for the two special issues. Out of the cumulative total of 43 authors, 65% were social scientists, and 33% were medical scientists. Five of the six review papers that were conceptualized specifically for the special issues were co-authored by multi-institutional and multi-disciplinary teams. Two of these had authors based in Ghana.

**Table 5:** Regional participation in Partnership Journal Special Issues

	**#papers**	**#authors**	**Africa§**	**Asia**	**Europe**	**US**
Globalization and Health SI (2010)	8	31	7	1	17	6
Ghana Medical Journal SI (In Press)	10	20	12	–	7	1
Total	18	46*	19	1	20	5

*5 partners contributed to both Special Issues(SIs); §Cameroon, Ghana, Nigeria, Kenya; Malaysia; Netherlands, Denmark; UK.

**Table 6:** Disciplines of contributing authors to Partnership Journal Special Issues

	**# papers**	**# authors**	**Social sciences**	**Humanities**	**Medical sciences**
Globalization and Health SI (2010)	8	31	25	1	5
Ghana Medical Journal SI (In Press)	10	20	9	–	11
Total	18	46*	31**	1	14***

*5 partners contributed to both SIs; **3 partners published in both SIs; ***2 partners published in both SIs.

The British Academy commissioned and published a research report based on the 2009 Accra Conference [10]. The report was launched at the LSE, on 1st June 2011, to stimulate interest in and discussion of Africa’s chronic disease problems ahead of the UN High Level Meeting on NCDs in September 2011 [49]. A follow-up launch was held in Accra in April 2012.

The 2010 Malaysia meeting led to the development of two new special issues that aimed to address the challenge of NCDs in low and middle-income countries. A *Globalization and Health* issue titled “Chronicity” and Chronic Health Conditions: Implications for Health and Health Care is in press.

This issue was guest edited by organisers of the Monash conference who were based in Africa, Asia, and the UK. A second special issue in *Ethnicity and Health* is titled Culture, Ethnicity and Chronic Conditions: a Global Synthesis. This issue is expected to be published in 2013 and is guest edited by partnership members based in Africa, Asia, the UK and the US.

## An evaluation of the partnership’s achievements and challenges

### Building a successful research partnership: enabling factors and barriers

Table 7 presents an evaluation of our achievements using the Maselli, Lys and Schmid (2005) criteria [5]. We outline the enabling factors and barriers to achieving our four goals in order to highlight why and how our partnership worked.

**Table 7:** Matching Partnership Goals to Criteria for building communities of research excellence

**Maselli, Lys and Schmid (2005)**	**Partnership achievement(s)**	**Enabling Factors**	**Barriers**
1. Decide on objectives together	Yes	A working relationship between the lead partners and the core working group based on trust, respect and openness. Good communication access (e.g. email, phone, annual meetings).	None
2. Build up mutual trust	Yes	Pre-existing relationships between applicants on original grant. Trust, respect and openness, key aspects of these relationships.	None
3. Share information, develop networks	Yes	Good communication channels between partners (see 1 and 2).	Partnership website was developed but not fully functional in the first two years due to limited administrative support (see 4).
		Openness and flexibility to involve new members and especially postgraduate students.	
		Funding for annual meetings created platform for sharing information and developing networks.	
4. Share responsibility	Yes	Shared commitment by core working group members (see 1,2 and Box 3)	Limited funding and capacity for administrative and management support. The coordination of tasks and activities was the responsibility of the lead partners who had full-time academic responsibilities. As lead UK partner transitioned from postdoctoral fellowship status to a full-time lecturing position, the time and capacity to engage in increased administrative tasks diminished.
5. Create transparency	Yes	Informal and flexible communication approach. Commitment by lead partners to disseminate important information, e.g. levels of engagement, meetings, opportunities for publications, calls for grant proposals.	None
6. Monitor and evaluate collaboration	Yes	Reflexivity built into project because of BA reporting processes.	Lack of clear indicators to measure some goals (e.g. Goal 2).
		Flexibility and simplicity of BA reporting processes.	Limited funding and a lack of administrative support affected the monitoring and evaluation of external processes (e.g. the impact of policymakers’ participation in partnership events on policy development)
7. Disseminate the results	Yes	Funding available for annual multi-stakeholder meetings.	Monitoring and Evaluation indicators were not systematically outlined at the outset of the project. This affected the evaluation of goal 2 (the translation of research dissemination into policy development).
		In-kind support from hosting institutions (e.g. fee waivers for cost of venue, logistical assistance with publicity).	
		Access to journal editors through core working group members’ networks.	
8. Apply the results	Yes	Access to journal editors enabled proceedings of partnership meetings to reach a wider international audience	Failure to secure funding prevented the development of collaborative research in the first three years.
		Creative strategies enabled the development of pilot projects funded by northern institutions	
		Access to funding (competitive grants and seed funding) and the administrative capacity of northern partners’ institutions enabled the development of collaborative research projects	
9. Share profits equitably	Partially	A spirit of openness and inclusiveness enabled collaboration between northern and African members as well as senior academics and postgraduate researchers. Profits (mainly publications, travel opportunities) were shared by core working group members.	Lack of funding affected the sharing of some profits (e.g. international travel to partnership meetings were not available to all members (see Table 1)
10. Increase research capacity	Partially	Access to northern institutional seed funding	Limited funding during first 3 years of partnership.
11. Build on achievements	Yes	Access to in-kind support by African institutions	Multiple responsibilities shared by few core working group members.
		Commitment from core working group.	Lack of administrative and management capacity is likely to undermine grant proposal writing by African partners, especially partners outside Ghana.
		Access to seed funding from northern institution to support US-Ghana research collaboration	Lack of funding may undermine our medium term goal to establish regular international conferences on chronic conditions in Africa.
		Access to in-kind support from African institutions	
		Access to a competitive grant to support Ghana- Europe research collaboration	

Jones et al. [2] suggest that partnerships work when the following factors are present: relations of mutual trust and respect; responsiveness of northern partners to southern partner demands; partnership structures do not add to cumbersome donor requirements; the local context is understood; the development of projects build on existing capacities. The majority of the enabling factors we identified map onto the factors identified by Jones et al. [2]. The relationships between working group members were built on trust, mutual respect and openness. Our activities were characterized by operational flexibility, which was supported by the flexibility and simplicity of The British Academy’s reporting process. We applied creative strategies to maximize our minimal resources, for example in negotiating in-kind support from southern partner institutions and accessing financial support from northern partner institutions.

Our barriers were material and conceptual. We lacked funding to conduct research, to train postgraduate students and to evaluate some of our goals. We also lacked administrative support: the majority of tasks had to be carried out by a small group of partnership members who had full-time academic responsibilities. Conceptually, the partnership goals were over-ambitious; furthermore we did not develop clear indicators to measure the success of some of our goals. For example, we did not build appropriate monitoring and evaluation processes into our policy goal. Therefore while we were able to reach policymakers across three continents through our research dissemination platform, it is difficult to make any claims about the transfer of knowledge into policymaking.

**Figure 2:**
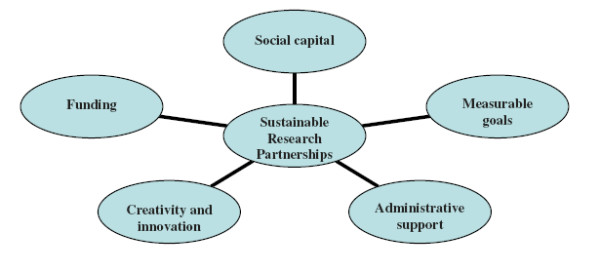
Five key ingredients for sustaining research partnerships.

We identified five key ingredients required to sustain our partnership (see Figure 2) that may be applicable to research partnerships generally:

1. Social Capital: A basic definition of social capital is the shared understandings, values and links individuals and groups share that engender trust and collaboration. Theorists make distinctions between bonding capital (‘trusting and co-operative relations between members of a network who are similar in a socio-demographic sense’), bridging capital (‘relations of respect and mutuality between people who are dissimilar’) and linking social capital (norms of respect and networks of trusting relationships between people who are interacting across explicit, formal, or institutionalized power or authority gradients in society’) [50]. The importance of trust and mutual respect within partnerships (bonding capital) is emphasized by the majority of reflexive accounts of sustainable partnerships. Our partnership thrived, despite funding challenges, because these elements were present within our core working group. We also had the added advantage of accessing support from our funder, The British Academy, and institutions of our northern partners (linking capital) which strengthened aspects of advocacy activities and postgraduate support, respectively (see Table 7).

2. Measurable goals: Goals have to clearly be conceptualized, realistically costed and measurable in order to monitor and evaluate the relationships between inputs, outputs and outcomes. It helps to develop indictors for monitoring and evaluating goals at the inception stage. Beyond their utility in tracking progress, indicators also enable activities, responsibilities and expected outcomes to be transparent to all partners.

3. Administrative support. Administrative support is necessary for non-technical aspects of partnership activities. Activities like organizing meetings (whether face to face or online), writing reports, searching for grant proposals and developing grant proposal budgets can be time consuming and cumbersome. They add on extra responsibilities that stretch the capabilities and commitments of partnership members.

4. Creative and innovative strategies: Openness to new ways of using existing resources (within the group) and to securing additional resources (within and outside the group) can propel a poorly-resourced research partnership forward. In our experience, creative strategies are collectively understood, legitimized and supported when partnerships have bonding social capital and strategies can be successful when partnerships develop linking social capital with external networks and groups with relevant material resources.

5. Funding. For small scale partnerships to transition from the grant stage to the integrative stage, funding is required. The primary route is for partners to access competitive grants if they have the appropriate capacity to develop and submit proposals. Other routes might be to access non-competitive grants, such as seed funding from existing funding and academic networks. A recent alternative has been offered by The Nairobi Report, an influential document on building capacity in African universities that was produced through consultation with UK and African academics [6]. The report advocates “the need for a more integrated research funding system that can provide ‘a ladder’ for the progression of good collaborative research teams from small-scale intensive projects to test initial research ideas and methodologies to large scale projects that can provide rigorous evidence” (p. vii). We are strongly aligned to this view. For successful small-scale research partnerships that focus on important research problems that are marginalized by local and international policymakers and funders, the progress from small-scale (grant stage) to large scale projects (integrative stage) must be actively supported by the initial funders.

### The collaborative continuum: moving from the transactional stage to the integrative stage

Funding from the British Academy ended in 2010 and we no longer have funding tied solely to the partnership. Thus, in terms of the collaborative continuum (Figure 1) we are at the transactional stage in which members of the core working group are combining resources—time, research expertise, access to grants—to achieve our goals. To move from the transactional stage to the integrative stage, where resources are merged to generate a new identity [24], we must address at least two challenges.

The first challenge relates to institutional support and its associated administrative support. Our institutional arrangements have changed, with LSE Health and RIPS, rather than Cambridge and NMIMR, providing the institutional support role. These changes have occurred for three reasons. First because funding tied to Cambridge ended. Secondly, because the UK principal partner (AdGA), formerly based at Cambridge, joined RIPS in 2009, with a visiting fellowship at LSE Health in the same year. Thirdly, because the LSE Health directorship, has supported the initiative since 2009, and committed funding and in-kind contributions to partnership activities. The critical issue is whether this institutional arrangement is the most appropriate for moving the partnership into the integrative stage. Partnership activities in the two years following the end of BA funding have been supported primarily by core working group members based in four institutions: RIPS, LSE Health, University of Amsterdam and NYU. This arrangement whereby the partnership retains an identity as ‘a community of practice’ [6] with access to support from southern and northern institutions has worked in concrete ways. It has, for example, created the opportunity for partnership members to collaborate on research (Goal 1) and to train postgraduates (Goal 3).

A major European Union (EU) grant was secured by a consortium led by CA in 2011. The project—titled Risk of Obesity and Diabetes among African Migrants (RODAM)—focuses on the complex interplay between environment, lifestyle, (epi)genetic as well as social factors in type II diabetes and obesity among one homogenous migrant population (i.e. Ghanaians) living in Germany, the Netherlands, the UK and their counterparts living in rural and urban Ghana. The RODAM project will generate relevant results that will ultimately guide intervention programmes and will provide a basis for improving diagnosis and treatment among sub-Saharan African migrants in Europe as well as in their counterparts in Africa and beyond. The consortium includes 5 members of the core working group. Three PhD studentships will be available for students at RIPS and the Kumasi Centre for Collaborative Research (KCCR), Kwame Nkrumah University of Science and Technology. In addition, NYU has provided seed funding for collaborative research between NYU and UG. Two core working group members are collaborating on cardiovascular disease management projects and building on existing population-based research at RIPS. Graduate support is built into these projects, with the creation of 4 MPhil studentships and 1 PhD studentship at RIPS.

The partnership also requires administrative and management support. To date the majority of responsibilities and tasks have been carried out by a limited number of core working group members who are full-time academics. This has often affected the timely development and delivery of partnership activities. As the partnership moves into the integrative stage, a budget for administrative and management support must be built into our funding applications.

The second challenge relates to the kind of ‘identity’ the partnership should develop in the long-term. Our original set of goals was over-ambitious given our limited funding. In the absence of partnership-specific funding, a prudent strategy might be to focus on the two goals we have successfully achieved: creating a platform for chronic disease research dissemination, including the support and dissemination of postgraduate student research. While the RODAM and NYU-UG projects offer concrete opportunities for core working group members to collaborate on to achieve research and postgraduate student training, these projects are not strictly owned by the partnership.

In the medium term (2012–2015), resources from core working group members based at RIPS, Amsterdam and NYU are likely to push the partnership into the integrative stage. This sub-group continues to work together on grant applications. We have publishing projects in progress until 2013. The partnership also continues to receive queries for membership from established researchers and postgraduates from Africa, UK and US, suggesting that we provide an important function in the arena of African-centred chronic disease research. As our major achievement was making NCD research in Africa more visible to an international audience, we aim to build on this by establishing a biennial *International Conference on Chronic Conditions in Africa*.

## Conclusions

Our research partnership set out to develop a model for NCD research, postgraduate training and support, policy development and advocacy over a three-year period. We achieved major successes with very limited funding. Key successes included creating greater visibility for NCD research in Africa and supporting African and European postgraduate students by offering them publication opportunities. Our meetings in Ghana, UK and Malaysia also created the space for researchers to network with, and disseminate their research to, policymakers.

Our collaborative research goal was not successfully realized during the funded life of the project due to limited funding and our failure to secure additional funding. We were also limited by the lack of administrative support. However the trust, respect and openness between the core working group and shared passion for and commitment to developing research solutions for Africa’s chronic disease burden shaped practical and creative responses to the challenges we experienced. These elements of bonding social capital were crucial to the success of our partnership during the BA funded phase and to its sustainability beyond the funded phase.

The importance of funding cannot be underestimated, however. Over the last two years, the ability of our partnership to increase its research capacity and build on its achievements has been possible through funding accessed by partners in northern institutions. We endorse the recent call for integrated funding systems that provide a ladder for the progression of ‘good collaborative research teams’ from small-scale projects to large scale projects [6]. For successful small-scale projects based on important health and developmental problems that are marginalised by local and international policymakers and funders, it is essential that the progress from small-scale to large scale projects is actively supported by the initial funders. The support can be provided through extra targeted funding the initial funders can offer themselves or secure from their funding networks. The support can benefit medium term activities such as the preparation of further grant proposals and long-term activities such as administrative tasks, which are crucial for projects to progress from the grant stage to the integration stage.

Beyond the specific challenges of sustaining a chronic disease research partnership, we identified and discussed the importance of social capital, measurable goals, administrative support, creative and innovative strategies and funding as five key ingredients that are essential for sustaining research partnerships generally.

## Abbreviations

AMC: Amsterdam Medical Centre; APHRC: African Population and Health Research Centre; BA: The British Academy; BMJ: British Medical Journal; CVD: Cardiovascular Disease; EU: European Union; GAAS: Ghana Academy of Arts and Sciences; GHI: Global Health Initiatives; ISAAC: International Study of Asthma and Allergy in Childhood; LSE: London School of Economics and Political Science; MDG: Millennium Development Goals; MHaPP: Mental Health and Poverty Project; NCD: Non-communicable Disease; NMIMR: Noguchi Memorial Institute for Medical Research; NOW: Netherlands Organisation for Scientific Research; NYU: New York University; PI: Principal Investigator; RIPS: Regional Institute for Population Studies; RS: Royal Society; UG: University of Ghana; UK: United Kingdom; UN: United Nations; US: United States; WHO: World Health Organization.

## Competing interests

The authors declare that they have no competing interests**.**

## Authors’ contributions

AdGA drafted the article. CA and EP contributed to subsequent revisions. All authors read, discussed and approved the final version.

## Authors’ information

AdGA is a social psychologist with a primary interest in chronic illness experiences and mental health in African communities, with a focus on diabetes experiences in Ghana. DKA is a medical anthropologist with research interests in access to medicines and health financing in Africa. EP has a background in health sciences and public health. Her research has focused on access to and quality of women’s health services in the UK and low and middle income countries. GO is a cardiovascular physician and researcher. His research has focused on cardiovascular disease interventions among minority ethnic groups in the United States and in continental Africans. PA is a public health researcher working across the disciplines of medical anthropology and epidemiology. Her research has focused on the health of populations marginalised by gender, ethnicity, disability and disease. CA is an epidemiologist and public health scientist. His research focuses on cardiovascular diseases among ethnic groups and in low- and middle-income countries.
